# Simple and Accurate HPTLC-Densitometric Method for Quantification of Delafloxacin (A Novel Fluoroquinolone Antibiotic) in Plasma Samples: Application to Pharmacokinetic Study in Rats

**DOI:** 10.3390/antibiotics9030134

**Published:** 2020-03-23

**Authors:** Prawez Alam, Muzaffar Iqbal, Essam Ezzeldin, Nasr Y. Khalil, Ahmed I. Foudah, Mohammed H. Alqarni, Faiyaz Shakeel

**Affiliations:** 1 Department of Pharmacognosy, College of Pharmacy, Prince Sattam bin Abdulaziz University, Al-Kharj 11942, Saudi Arabia; p.alam@psau.edu.sa (P.A.); a.foudah@psau.edu.sa (A.I.F.); m.alqarni@psau.edu.sa (M.H.A.); 2 Department of Pharmaceutical Chemistry, College of Pharmacy, King Saud University, Riyadh 11451, Saudi Arabia; esali@ksu.edu.sa (E.E.); nkhalil@ksu.edu.sa (N.Y.K.); 3 Bioavailability Unit, Central Laboratory, College of Pharmacy, King Saud University, Riyadh 11451, Saudi Arabia; 4 Department of Pharmaceutics, College of Pharmacy, King Saud University, Riyadh 11451, Saudi Arabia; fsahmad@ksu.edu.sa

**Keywords:** antibiotic, Delafolxacin, HPTLC, pharmacokinetic study, validation

## Abstract

Delafloxacin (DLX) is a recently-approved fluoroquinolone antibiotic, which is recommended for the treatment of “acute bacterial skin and skin structure infections”. A thorough literature survey revealed only a single published method for the estimation of DLX using UPLC-MS/MS technique in biological samples. There is no high-performance thin-layer chromatography (HPTLC) method has been reported for the estimation of DLX in dosage forms and/or biological samples. Therefore, a selective, sensitive, rapid and validated HPTLC-densitometry technique has been used for the estimation of DLX in human plasma for the first time. HPTLC quantification of DLX and internal standard (IS; gatifloxacin) was carried out on glass coated silica gel 60 F_254_ HPTLC plates using the ternary mixture of ethyl acetate:methanol:ammonia solution 5:4:2 (%, *v/v/v*) as the mobile phase. Densitometric detection was done at 344 nm. The R_f_ values were recorded as 0.43 and 0.27 for the DLX and the IS, respectively. The linearity range of DLX was obtained as 16–400 ng/band. A simple protein precipitation method was used for the extraction of analyte from plasma using methanol. The proposed HPTLC technique was validated for “linearity, accuracy, precision, and robustness”. The proposed HPTLC technique was successfully utilized for the assessment of pharmacokinetic profile of DLX in rats after oral administration. After oral administration, the peak plasma concentration of DLX was obtained as 194.19 ng/ml in 1 h. The proposed HPTLC method could be applied in study of pharmacokinetic profile and therapeutic drug monitoring of DLX in clinical practice.

## 1. Introduction

Acute bacterial skin and skin structure infections (ABSSSIs) and community-acquired respiratory tract infections (CARTIs) are the most common infections which require hospitalization of patients for the treatment [1,2]. Delafloxacin (DLX), which is a new fluoroquinolone antibiotic, recently approved for the treatment of both ABSSSIs and CARTIs by USFDA and for ABSSSIs only by EMA [3,4,5]. It has been found active against major pathogens responsible for ABSSSIs and CARTIs [6,7]. Unlike other fluoroquinolones, chemical structure of DLX is different due to absence of a basic group next to the fluorinated ring. This change contributes to the weakly acidic properties of DLX and it has better penetration and accumulation to infected cells and bacteria. DLX is rapidly absorbed after oral administration as its peak plasma concentration is reached within 1–2.5 h [8,9]. Compared with other fluoroquinolone antibiotics, the absolute bioavailability of DLX has been reported as low (58.8%) which could be due to its weak solubility in water [10]. For the treatment of ABSSSIs and CARTIs, the frequent oral dosing of DLX is required [7,11]. However, frequent oral administrations are often discouraged due to patient compliance reason which may lead to resistance for fluoroquinolones in the treatment of ABSSSIs and CARTIs [12]. A thorough literature survey revealed a single ultra-performance liquid chromatography-mass spectrometry/mass spectrometry (UPLC-MS/MS) technique for the determination of DLX in rat plasma and rabbit aqueous humor [10]. Some researchers reported the assay for the assessment of pharmacokinetic profile of DLX in healthy human volunteers and some diseased patients [11,13,14]. Nevertheless, these researchers had not disclosed any information about the method development and validation of these assays. To the best of the authors’ knowledge, not a single high-performance thin-layer chromatography (HPTLC) method has been reported for the estimation of DLX in pharmaceutical dosage forms and/or biological samples such as rat or human plasma. Although, UPLC-MS/MS method was developed and validated well for the determination of DLX in biological samples, but the cost and maintenance of UPLC-MS/MS apparatus is expensive [10,15]. In addition, highly-skilled technical personnel are required to operate such apparatus and hence not commonly available in most of the laboratories. Moreover, in previous UPLC-MS/MS assay, sample extraction was performed by liquid–liquid extraction, since matrix effects produced by protein precipitation was high and was not acceptable. Therefore, an economical and convenient methodology is generally required for the analysis of drugs in biological samples with reasonable analysis time. Such methodologies must be capable to provide the lower limit of quantitation (LLOQ) of drugs and suitable for routine analysis. The recommended dose of DLX is 300–450 mg in adults’ patients and therefore the LLOQ wouldn’t be a challenging task during pharmacokinetic analysis. Due to the advancements in the stationary phases and the inclusion of densitometers as detection apparatus, HPTLC methodologies achieve good precision and accuracy compared with HPLC [16,17]. Nevertheless, the application of HPTLC for the estimation of drugs in biological samples is limited [18,19,20]. The objective of this work was to develop and validate a simple and reliable HPTLC technique for the estimation of DLX in human plasma. Compared to previous method, the sample extraction was done by protein precipitation by using methanol which is simple and less tedious process. The developed HPTLC technique was successfully applied in pharmacokinetic assessment of DLX in real plasma samples of rats. 

## 2. Materials and Methods

### 2.1. Chemicals and Reagents

DLX (purity ˃ 98%) was procured from “Beijing Mesochem Technology Co. Ltd. (Beijing, China)”. Internal standard (IS; gatifloxacin; purity ˃ 99%) was obtained from “Sigma Aldrich (St. Louis, MO, USA)”. HPLC grades of methanol and ethyl acetate (EA) were obtained from “Fisher Scientific Ltd. (Leicestershire, UK)”. All aqueous solutions were prepared using Milli-Q water which was collected from “Milli-QR GradientA10R (Millipore, Moscheim Cedex, France)”. 

### 2.2. Instrumentation and Analytical Conditions

HPTLC-densitometry estimation of DLX was conducted on “10 × 20 cm glass coated silica gel 60 F_254_ plates HPTLC plates (E-Merck, Germany)”. Samples were applied to the TLC plates as 6 mm bands using a “CAMAG Automatic TLC Sampler 4 (ATS4) sample applicator (Geneva, Switzerland)” fitted with a “CAMAG microliter syringe”. A constant application rate of 150 nl/s was used for this estimation. Linear ascending development of the plates to a distance of 80 mm was carried out using EA:methanol:ammonia solution (5:4:2, % *v/v/v*) as the mobile phase in a “CAMAG Automatic Developing Chamber 2 (ADC2)” which was saturated previously with the mobile phase vapor for 30 min at 22 °C. The densitometric-scanning was achieved in the absorbance-reflectance mode at *λ*_max_ = 344 nm after scanning between 200–400 nm using a deuterium lamp. The slit dimensions were 4 mm length and 0.45 mm width, with a scanning rate of 20 mm/s. Each track was scanned in triplicates manner and baseline correction was used. The “win CATS software (version 1.4.2, CAMAG)” was used to control the operative parameters during the whole analysis. 

### 2.3. Sample Preparation

Accurately weighed amounts of each of DLX and IS working standards was dissolved in methanol in order to obtain the stock solutions of 500 µg/mL. The stock solution of DLX was further diluted with 50% methanol solution to prepare the working standard for calibration curves (CCs) and quality control (QC) samples. Then, the working standards of CCs and QC samples were spiked to DLX free human plasma samples to achieve the CCs range of 16–400 ng/band and QC samples. The stock solution of IS was also diluted with 50% diluted methanol solution to obtain the working standard of 1000 ng/mL IS solution. All aqueous solutions were stored in refrigerator while spiked plasma samples were stored in deep freezer at −80 °C till further analysis. 

### 2.4. Sample Extraction

Before applying extraction procedures, all samples (i.e., CCs, QCs, and real rat plasma samples) stored at −80 °C were kept for 2 h at 22 °C for thawing and vortexed for about 30 s before sample preparation. In a fresh 2.0 mL centrifuge tube, 200 µL aliquot of all plasma samples including blank was taken and 15 µL of IS (1000 ng/mL) was added. The samples were vortexed again for 30 s and proteins were precipitated using 385 µL of methanol. The samples were again gently vortexed for about 1.0 min. The samples were centrifuged for about 10 min at 11,000× *g* at 4 °C. After centrifugation, the supernatant was taken into a fresh tube and 8 µL of sample was applied on TLC plate for the estimation of DLX and IS.

### 2.5. Method Validation

The proposed HPTLC technique was validated according to FDA guideline for bioanalytical procedures [21]. The method was validated in terms of “selectivity, sensitivity, linearity, accuracy, precision, recovery, and stability”. 

#### 2.5.1. Selectivity and Specificity

The specificity of the method was determined by comparing the spectra, peak area and R_f_ values of spiked plasma samples bands with that of DLX and IS. The selectivity of the method was estimated by comparing the HPTLC response in blank plasma matrix at the R_f_ value of DLX and IS with plasma spiked with LLOQ (i.e., 16 ng/band). Six different batches of blank human plasma were spiked with LLOQ concentration of DLX (16 ng/band) and IS (100 ng/band). DLX and IS were separated by a protein precipitation method and quantification was carried out by the proposed HPTLC technique [10,21]. 

#### 2.5.2. CCs and Linearity

The CCs (*n* = 6) were obtained in spiked human plasma at six different concentrations in the range of 16–400 ng/band. The linearity was determined by constructing the CC between the peak area ratios of DLX to IS and concentration of DLX. The data of CCs was treated and analyzed using least squares linear regression analysis. The reliability of CC was evaluated by determining the accuracy and precisions of the CC at six different concentrations of DLX. The variation in accuracy and precision of CC has been recommended as ≤15% [11]. The minimum concentration of DLX in CC was considered as LLOQ which had a signal-to-noise ratio of ≥5 in comparison with blank plasma [15,21]. 

#### 2.5.3. Sensitivity

The sensitivity of HPTLC assay was estimated in terms of “limit of detection (LOD) and limit of quantification (LOQ)”. The LOD was calculated as 3.3 σ/S and LOQ was calculated as 10 σ/S. Here, σ is the intercept and S is the slope of the CC [10,21]. 

#### 2.5.4. Precision and Accuracy 

Precision and accuracy of HPTLC assay were obtained in human plasma at three different QC concentration levels i.e., LQC (16 ng/band), MQC (200 ng/band) and HQC (400 ng/band). The intra-day precision and intra-day accuracy were evaluated on the same day (*n* = 6). However, the inter-day precision and inter-day accuracy were evaluated on three different days (*n* = 18). The uncertainty in the precision has been recommended as ˂20% for LQC sample and 15% for the other samples [21]. However, the uncertainty in accuracy should be within the limit of ±20% for the LQC sample and ±15% for the other QC samples [10,21].

#### 2.5.5. Robustness

The robustness of HPTLC assay was determined by introducing small deliberate change in the analytical conditions. In this study, the small deliberate change in the mobile phase was done and the MQC (200 ng/band) was quantified using proposed HPTLC method. Recovery and precision were calculated for the determination of the robustness [17].

#### 2.5.6. Recovery Studies

The recovery of HPTLC assay was also evaluated at 16 ng/band, 200 ng/band and 400 ng/band concentrations. The recovery of DLX from human plasma was determined by comparing the peak area ratio of plasma spiked with DLX prior to extraction with those spiked with DLX after the extraction [15,21]. 

#### 2.5.7. Stability Study

The stability analysis of DLX was performed in human plasma by estimating 16 ng/band (LQC) and 400 ng/band (HQC) concentrations (*n* = 6) of DLX under different storage conditions. All stability studies were conducted and evaluated by freshly prepared CC of DLX. The short-term stability of DLX was determined by processing and estimating LQC and HQC samples after 12 h storage at 22 °C i.e., bench-top stability. Freeze-thaw stability of DLX was estimated after freezing the spiked LQC and HQC samples at −80 °C and thawing at 22 °C for three cycles. Long-term stability of DLX was determined by estimating the spiked LQC and HQC samples stored at −80 °C for about 30 days. The standard and working QC samples of DLX and the IS were also evaluated for their stability at 22 °C for 12 h and at refrigerator temperature (below 8 °C) overnight. The uncertainties in LQC and HQC samples within the limits of accuracy (±15%) and precision (≤15%) suggested the stability of the method [17,21].

### 2.6. Application of HPTLC Assay to Pharmacokinetic Study in Rats

Since our affiliation is academic organization and does not have a hospital or clinical pharmacology unit and also due to complexity of ethical issues pertaining to administer the drugs in human subjects, the study in patients or normal volunteers could not be taken up. But, developed method was applied to a preliminary pharmacokinetic study of DLX in rats. Before application in rats, a partial method validation in term of accuracy and precision were performed in blank rat plasma samples. Male Wistar Albino rats (weighing 200–250 g; *n* = 6)” were obtained from the “Animal Care Centre, College of Pharmacy, King Saud University, Riyadh, Saudi Arabia”. The protocol for these studies was reviewed and approved by “Research Ethics Committee (Ref. no. KSU-SE-19-27) of College of Pharmacy, King Saud University, Riyadh, Saudi Arabia”. The experiments were performed as per the guideline for “Animal Care and Use Committee of King Saud University”. The rats were kept in polypropylene cages. All the rats were given overnight fasting before starting the experiments. After overnight fasting, all the animals were administered with DLX (20 mg/kg oral suspension in sodium carboxymethyl cellulose). The blood samples (approximately 0.5 mL) were withdrawn from the retro-orbital plexus into heparinized tubes at different time intervals (0, 0.5, 1, 1.5, 2, 4, 6, and 12 h). Plasma was separated from each blood sample by centrifuging the blood at 4500 g for 8 min and frozen at −80 °C till further analysis. The pharmacokinetic parameters such as peak plasma concentration (C_max_), time to reach C_max_ (T_max_) and area under curves from time 0 to t (AUC_0-t_) were determined using “MS Excel program 2010”.

## 3. Results and Discussion

### 3.1. Optimization of Analytical Procedures

**Figure 1:**
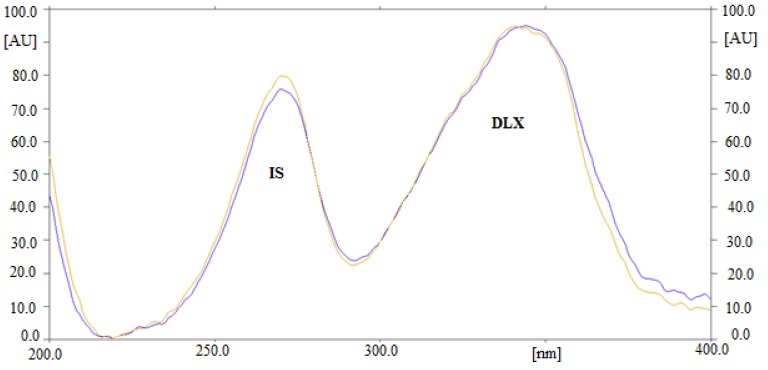
Overlaid UV absorption spectra of Delafloxacin (DLX) and internal standard (IS).

**Figure 2:**
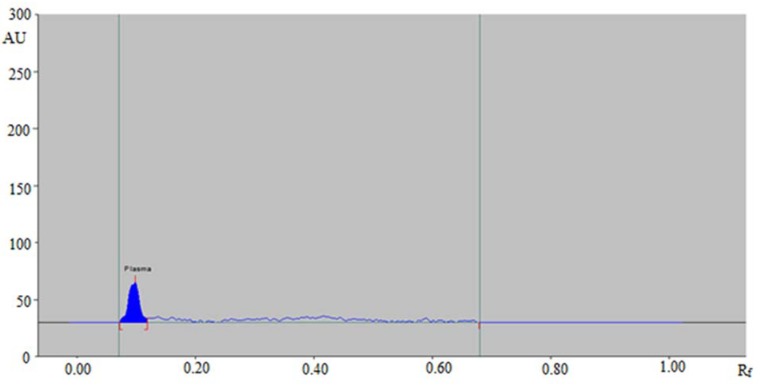
High-performance thin-layer chromatography (HPTLC) densitogram of blank human plasma sample.

The estimation of DLX in human plasma could be adjusted by changing the composition of the mobile phase. Initially, various solvents like hexane, ethyl acetate (EA), methanol, ethanol, acetonitrile, and chloroform were attempted to develop HPTLC method. The results indicated that when DLX was estimated using methanol and EA, the R_f_ value was shifted toward the upper side (more than 0.8). Different mixtures of EA and methanol were also tried. A sharp and well-resolved peak of DLX at R_f_ = 0.43 ± 0.01 was achieved when the ratio of EA: methanol: ammonia solution was 5:4:2 (%, *v/v/v*). For the optimization of the detection wavelength for the analysis of DLX, different solutions of DLX were prepared and their UV absorption spectra were recorded. Upon evaluation of their overlaid spectra (Figure 1), it was observed that the DLX presented considerable absorbance at 344 nm and therefore it was selected as the analytical wavelength for further analysis. Due to recovery and extraction issues, bioanalytical methods for the estimation of drugs in plasma utilize the use of an internal standard (IS) [10]. Therefore, many drugs were studied as IS in order to find out the best one. Finally, gatifloxacin was found as the ideal one, as its wavelengths were close to that of DLX. Also, a good resolution between DLX (0.43 ± 0.01) and gatifloxacin (0.27 ± 0.02) was obtained under the proposed analytical procedures (Figure 2). Hence, gatifloxacin was selected for all the subsequent experiments as an IS.

### 3.2. Method Validation

The proposed HPTLC assay was validated as per the FDA recommendations for bioanalytical procedures [21]. All results are presented in percentages, where n represents the number of replicates. The significance was considered at a 5% level of significance. The proposed HPTLC technique was validated in terms of the following parameters.

#### 3.2.1. Selectivity and Specificity

The “specificity and selectivity” of the method was studied as per procedure described in materials and methods section. Good correlation was observed between analyte and spiked human plasma spectra, peak area, and R_f_ values. It was noted that the analyte was extracted completely from plasma without any possible appearance of plasma constituent peaks at the R_f_ values of DLX and IS (Figure 2). These results indicated that the developed HPTLC method was selective and specific for the estimation of DLX in biological samples.

#### 3.2.2. CCs and Linearity

The CC was made between the peak area ratio of DLX to the IS and various concentrations of DLX in human plasma. The CC was regressed statistically using linear regression analysis and results are listed in Table 1. The CC_S_ of DLX presented a good correlation between peak area ratio and concentration. The correlation coefficient (r) and determination coefficient (r^2^) values for DLX were estimated as 0.9996 and 0.9992, respectively. The linearity range for method was recorded as 16–400 ng/band. The regression equation for CC of DLX in human plasma was observed as Y = 0.0091x + 0.7493, where Y is the area ratio of DLX to IS and x is the concentration of DLX. These results indicated that the developed HPTLC method is linear and suitable for the estimation of DLX in plasma samples.

#### 3.2.3. Sensitivity

**Figure 3:**
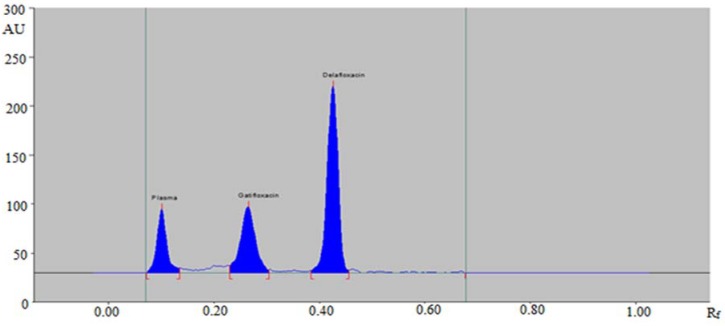
HPTLC densitogram of plasma sample spiked with DLX (100 ng/band) and (IS) (150 ng/band).

The sensitivity of the developed HPTLC method was determined in terms of “limit of detection (LOD) and limit of quantification (LOQ)”. The calculated values of “LOD and LOQ” are given in Table 1. The LOD and LOQ for the estimation of DLX in human plasma were calculated as 5.80 and 16.0 ng/band, respectively. The obtained values of LOD and LOQ indicated that the proposed HPTLC method is sensitive enough for the estimation of DLX in plasma samples. HPTLC densitogram of plasma sample spiked with DLX (100 ng/band) and (IS) (150 ng/band) are shown in Figure 3.

#### 3.2.4. Accuracy and Precision

The intra-day accuracy and precision of the developed HPTLC method were measured by studying the recovery and % RSD of DLX at three different concentrations: low quality control (LOQ), middle quality control (MQC) and high quality control (HQC) level (*n* = 6). However, the inter-day accuracy and precision were measured with same QC levels at *n* = 18. The results of accuracy and precision for DLX are presented in Table 2. The intra-day accuracy as % recovery of DLX was obtained as 96.26–102.53%. However, the inter-day accuracy was found as 93.37–103.57%. These results indicated the high accuracy of the proposed HPTLC method for the extraction of DLX. The intra-day and inter-day precision in terms of % RSD of DLX were estimated as 2.82–3.22 and 2.94–3.72%, respectively. The low values of % RSD indicated that the developed HPTLC method was precise for the estimation of DLX in human plasma.

#### 3.2.5. Robustness

For the evaluation of the robustness of the developed HPTLC method, small deliberate variations in mobile phase composition were introduced. The resulting data of robustness study is presented in Table 3. The influence of mobile phase composition on % recovery and % RSD was studied. The % recoveries (%) for robustness of DLX were estimated as 95.38–103.61% with % RSD values in the range of 2.82–3.60%. The higher values of % recoveries and lower values of % RSD recorded in this study indicated that the developed HPTLC method was robust for the estimation of DLX in human plasma.

#### 3.2.6. Recovery Studies

The results of recovery studies for DLX are presented in Table 4. The recoveries in the range of 93.31%–100.93% were recorded at three different concentrations of DLX. The high values of % recoveries indicated high efficiency of the method for the estimation of DLX in human plasma without interference from endogenous plasma constituents. 

#### 3.2.7. Stability Studies

Stability studies of DLX in human plasma were performed at four different storage conditions. The stability studies were carried out at LQC and HQC concentrations viz. “bench-top stability, refrigeration, long-term stability and freeze-thaw stability”. Results of stability evaluation are summarized in Table 5. The accuracy as % recoveries and precisions as %RSD for stability studies at different storage conditions were recorded as 94.25–106.56% and 1.18–6.34%, respectively. The studied plasma samples presented good stabilities in spiked plasma sample during sample preparation (bench-top stability) at 22 °C for at least 12 h and also after storage in refrigerator overnight below 8 °C. In addition, DLX was found to sufficiently stable after long-term and after three freeze–thaw stability studies. The results of this evaluation indicated the stability of the proposed HPTLC method.

### 3.3. Pharmacokinetic Study in Rats

**Figure 4:**
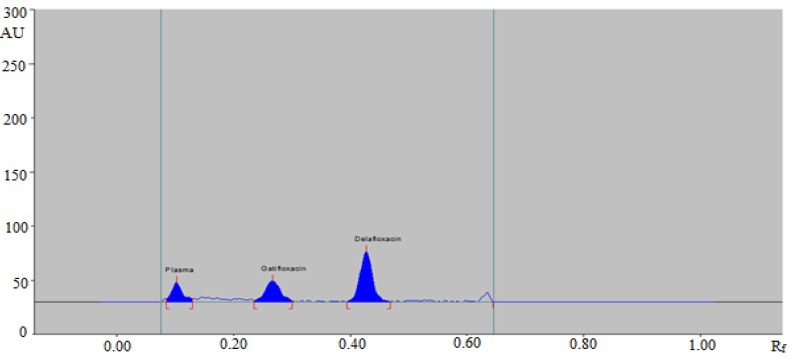
HPTLC densitogram of rat plasma obtained 1 h after oral administration of DLX in the presence of IS (150 ng/band).

**Figure 5:**
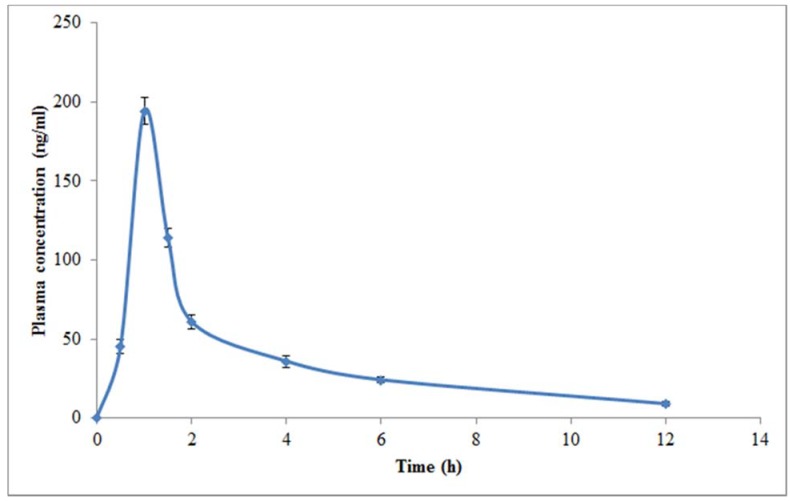
Plasma concentration-time profile curve of DLX after a single dose oral (20 mg/kg) administration in rats (mean ± SD; *n* = 6).

Pharmacokinetic studies were performed in rats due to its easy availability and relatively low variations in pharmacokinetics parameters. Hence, in order to study the validity of the method in real samples, the developed HPTLC assay was applied to study pharmacokinetic profile of DLX in healthy rats after oral administration of single dose of 20 mg/kg. The representative HPTLC densitogram of DLX along with IS after 1 h of oral administration in rats is shown in Figure 4. Three different pharmacokinetic parameters including C_max_, T_max_, and AUC_0-t_ are tabulated in Table 6. The mean value of C_max_ was estimated as 194.19 ng/mL after 1 h of oral administration. The mean value of AUC_0-t_ was recorded as 396.49 ng.ml/h. The plasma concentration-time profile graph of DLX after oral administration of DLX in rats is presented in Figure 4. The results presented in Figure 5 showed that DLX was absorbed rapidly after oral administration as its C_max_ was reached within 1 h of oral administration. The developed HPTLC method could be successfully applied to study pharmacokinetic assessment of commercial formulations of DLX. 

### 3.4. Literature Comparison

The developed HPTLC method for the determination of DLX in human plasma was compared with reported UPLC-MS/MS method [10]. The comparison results of present HPTLC method compared with reported UPLC-MS/MS method are tabulated in Table 7. Different validation parameters including “linearity range, sensitivity, accuracy, and precision” of the present HPTLC method were compared with the reported method. The reported UPLC-MS/MS method for the estimation of DLX was found better than present HPTLC method in terms of sensitivity, and wide linearity range but reported UPLC-MS/MS method was not better in terms of cost, sample extraction procedure, accuracy, and precision [10]. In addition, the present HPTLC method for the determination of DLX in plasma has been found more “simple, time saving and cost effective with good accuracy and precision” than reported UPLC-MS/MS method. Although, the present HPTLC method had low sensitivity than reported UPLC-MS/MS method, its sensitivity (LOD and LOQ values) was observed enough for the determination of DLX in plasma samples (due to its high dose strength) and therefore it overcomes the disadvantages of UPLC-MS/MS method such as “high cost and technical requirements”. Moreover, in previous reported LC-MS/MS method, liquid–liquid extraction was used for sample extraction from plasma sample due to poor recovery and high matrix effects with protein precipitation [10]. However, in this HPTLC method, matrix effect was not a matter of concerns, and therefore protein precipitation was used for sample extraction with high recovery. 

## 4. Conclusions

In the proposed research work, a simple, sensitive, selective, accurate and precise HPTLC method has been established for the first time for the determination of DLX in biological samples such as human plasma. The proposed HPTLC method introduces an innovative analytical idea, which is able to determine DLX without prior pretreatment from plasma. The statistical analysis indicated the absence of any interference from the endogenous plasma constituents. Therefore, the proposed HPTLC method could be applied successfully to study the pharmacokinetic and therapeutic drug monitoring of DLX in clinical practice with great efficiency. Furthermore, the developed HPTLC method was successfully applied for the study of pharmacokinetic profile of DLX in rat models. The developed HPTLC assay for the determination of DLX in plasma samples was found better than the reported UPLC-MS/MS method in terms of “cost, simplicity, accuracy, and precision”. 

## Figures and Tables

**Table 1:** Linear regression analysis data and quantitative parameters of DLX (*n* = 6). Limit of detection (LOD) and limit of quantification (LOQ).

Linearity Range (ng/Spot)	16–400
Regression Equation	Y = 0.0091x + 0.7493
Correlation Coefficient (r)	0.9996
Determination Coefficient (r^2^)	0.9992
Slope ± SD	0.0091 ± 0.0003
Intercept ± SD	0.7493 ± 0.0008
λ_max_ (nm)	344
R_f_	0.43 ± 0.01
LOD (ng/band)	5.80
LOQ (ng/band)	16.0

**Table 2:** Data for intra-day and inter-day precision and accuracy.

Conc. Added (ng/Band)	Intra-Day Assay (*n* = 6)			Inter-Day Assay (*n* = 18)		
	Conc. Found (ng/Band) ^a^	Precision (% RSD)	Accuracy (% Recovery)	Conc. Found (ng/Band) ^a^	Precision (% RSD)	Accuracy (% Recovery)
16	16.28 ± 0.46	2.82	101.75	14.94 ± 0.44	2.94	93.37
200	192.52 ± 5.94	3.08	96.26	192.97 ± 6.10	3.16	96.48
400	410.12 ± 13.24	3.22	102.53	414.28 ± 15.42	3.72	103.57

^a^ Mean ± SD.

**Table 3:** Robustness of HPTLC method at the concentration of 200 ng/band (*n* = 6).

Conc. (ng/Bnd)	Mobile Phase Composition (EA:Methanol:Ammonia)			Recovery (%) ± SD	RSD (%)	Rf
	Original	Used	Level			
200	5:4:2	4.9:4.1:2	+0.1	95.38 ± 2.69	2.82	0.41
		5:4:2	0.0	104.10 ± 3.11	2.98	0.42
		5.1:3.9:2	−0.1	103.61 ± 3.74	3.60	0.43

**Table 4:** Recoveries of DLX from spiked human plasma (*n* = 6).

Spiked Conc. (ng/Band)	Conc. Found (ng/Band)	Recovery (%) ± SD	RSD (%)
16	14.93	93.31 ± 3.13	3.35
200	197.41	98.70 ± 3.97	4.02
400	403.75	100.93 ± 5.22	5.17

**Table 5:** Stability evaluation of DLX at two different concentrations (LQC and HQC) (*n* = 6).

Stability	Nominal Conc. (ng/Band)	Conc. Found (ng/Band) ± SD	Precision (% RSD)	Accuracy (% Recovery)
Bench Top (12 h)	16	15.08 ± 0.85	5.63	94.25
	400	396.45 ± 4.68	1.18	99.11
Refrigeration (Overnight)	16	15.32 ± 0.96	6.26	95.75
	400	397.77 ± 5.25	1.31	99.44
Freeze Thaw (3 Cycles)	16	17.05 ± 1.02	5.98	106.56
	400	405.28 ± 6.91	1.70	101.32
Freezer at −80 °C (30 days)	16	17.65 ± 1.12	6.34	110.31
	400	401.13 ± 7.13	1.77	100.28

**Table 6:** Pharmacokinetic parameters of DLX after an oral administration of single dose (20 mg/kg) in rats (*n* = 6).

Parameters	Values (Mean ± SD)
C_max_ (ng/mL)	194.19 ± 8.20
T_max_ (h)	1.00 ± 0.01
AUC_0-t_ (ng.h/mL)	396.49 ± 8.53

**Table 7:** Comparison of the proposed HPTLC method with reported UPLC-MS/MS method for the determination of DLX in plasma.

Analytical Method	Linearity Range	Extraction Method	Detection Method	Cost	Accuracy (% Recovery)	Precision (% RSD)	Ref.
UPLC-MS/MS	3.5–5000 ng/ml	LLE ^a^	MS/MS	High	86.80–111.20	4.08–11.30	[10]
HPTLC	16–400 ng/band	PP ^b^	UV	Low	93.37–103.57	2.82–3.72	Present work

^a^ Liquid–liquid extraction; ^b^ Protein precipitation.
